# The Book World of Medicine and Science

**Published:** 1898-04-09

**Authors:** 


### THE BOOK WORLD OF MEDICINE AND SCIENCE.

Ethnological Studies amongst the North-west Central Queensland Aborigines. By W. E. Roth, B.A., M.R.C.S., &c. Pp. 199. Illustrations. (London, 1897: The Queensland Agent-General's Office.)

Dr. Roth, who has been for several years a medical officer in Queensland, has, in this volume, carefully collected a vast amount of information acquired in daily observation of the aborigines. The book is valuable, to the philologist and ethnologist alike, not only for its facts but for the expressions of opinion it contains. Of particular value is the chapter in which Dr. Roth records his discovery, amongst certain tribes, of an elaborate gesture language capable of expressing complex ideas. In another chapter Dr. Roth deals lucidly with the intricate system of genealogical nomenclature maintained by these aborigines, and points out how, in all probability, these systems and the dependent system of "tabu" have been devised to regulate the proper distribution of the total food supply. It is seldom that one finds in work, in this department of science, so careful and industrious an accumulation of facts, and so judicious a reserve of hypothesis, as in this of Dr. Roth's.

S. Maw, Son, and Thompson's Price List of Aseptic and Other Hospital Furniture.

This illustrated price list will be found to be very useful for those engaged in hospital or private practice. The latest improvements in operating tables, ward wagons, and aseptic tables are given. Fig. 1 is certainly the best form of operating table. The glass slabs which form the top of the table do not, as in most tables, fit into frames, but are made to overlap the frames, so that there are no angles or corners in which dust or dirt can collect. The table is fitted with Victor Horsley's head rest, which must now be considered a necessary addition to any modern operating table. The brackets for supporting plate glass shelves for lotion jars look very useful, and are inexpensive. The irrigators used at Bartholomew's and Middlesex are to be strongly recommended; they can be fixed to the roof or on a bracket, and are elevated by means of pulleys. The dressing wagon used at St. Mary's seems to be the best that can be obtained. The invalid bedstead might be of value in cases where the patient can only be moved with difficulty, particularly in some cases of paraplegia where frequent changes of linen are necessary. Tins for sterilising instruments, glass dressing trays, and ligature boxes are rather below the average in expense. Indiarubber post-mortem gloves are 6s. 6d. each.

A Bibliography of Medicine. Being the Sections Relating to that Subject in the Best Books and the Readers' Guide. By William Swan Sonnenschein. (Published by Swan Sonnenschein and Co., Paternoster Square. 1897.) Pp. 453.

This book would be valuable to some medical men for references. The scope of the work is, however, too wide to allow of adequate treatment. The literature in each branch of medical science has in the present day grown to such an enormous extent that a bibliography has become almost an impossibility. The most useful parts of the book are the references with regard to the mediæval history of medicine, such as the Black Death and Plague. The histories relating to the pioneers of medicine are given. The collected writings of the various nations, such as Arabic, Hindu, Roman, and Spanish, form an interesting subject. The modern books of reference are not nearly sufficient for practical purposes; some of the most important works are omitted.

The Physiology of Pregnancy and Child Birth, Anatomically Represented with Explanatory Text. By Arthur E. Giles, M.D., F.R.C.S., Ed.M.R.C.P. (Published by Baillière, Tindall, and Cox, 20 and 21, King William Street, Strand. George Philip and Son, 32, Fleet Street. Price 3s. net. Pp. 23.)

This book would be of use for midwives and nurses. The chief facts connected with child-birth are given in a concise manner, and could be easily understood by anyone. There is a coloured diagram showing the position of the fœtus in utero, and its relation with the surrounding parts. A few practical hints are given, such as the possible occurrence of umbilical hernia if a binder is not applied. The anatomy of the pelvic organs is described, and also that of the abdominal viscera. The fœtal circulation and the changes that take place at birth are ably demonstrated by a diagram. The ignorance of midwives is constantly coming before the notice of medical men; the infinite harm which may result from a lack of knowledge of their subject should be a sufficient guarantee of the sale of Dr. Giles' little book.

